# Accuracy of Digital Anthropometry During Pregnancy: A Longitudinal Study

**DOI:** 10.21203/rs.3.rs-7400356/v1

**Published:** 2025-09-30

**Authors:** Steven Heymsfield, Sophia Ramirez, Jasmine Brown, Ryan Yang, Marianna Deynzer, Gabriela de Oliveira Lemos, Cassidy McCarthy, Sara Dube, John Virostko, Isaiah Janumala, Amy Nichols, Rachel Rickman, Saralyn Foster, Michelle Cassidy, Elizabeth Widen

**Affiliations:** Pennington Biomedical Research Center; Pennington Biomedical Research Center; Pennington Biomedical Research Center; The University of Texas at Austin; Pennington Biomedical Research Center; Pennington Biomedical Research Center; The University of Texas at Austin; The University of Texas at Austin; The University of Texas at Austin; The University of Texas at Austin; The University of Texas at Austin; The University of Texas at Austin; The University of Texas at Austin; University of Texas, Austin

**Keywords:** Body Composition, Obesity, Nutritional Assessment, Pregnancy, Circumference

## Abstract

**Background/Objectives:**

Maternal anthropometric changes are rapid during pregnancy and reflect increments in maternal and fetal tissues. These dynamic changes in body composition and shape during pregnancy are associated with maternal and fetal outcomes and are often monitored with simple tools such as a flexible tape for quantifying selected circumferences. The current study aim was to evaluate the hypothesis that circumferential measures of maternal body size and shape acquired with a 3D-optical imaging system will agree closely and correlate significantly with ground-truth estimates made with a flexible tape by trained staff.

**Subjects/Methods:**

3D-optical scans and flexible tape measurements were acquired at 15-, 25-, and 35-weeks of gestation in 57, 41, and 35 participants, respectively. 3D avatars obtained at each time point were analyzed for waist at two sites, hip, mid-upper arm, mid-thigh, and calf circumferences. 3D-optical and flexible tape measurements were compared using linear regression analyses (R^2^s), mean absolute errors (MAEs), root-mean square errors (RMSEs), concordance correlation coefficients (CCCs), and Bland-Altman plots.

**Results:**

Overall, agreement between 3D and conventional anthropometric measurements were strong at all five anatomic sites (R^2^s, 0.63–0.97; p’s all < 0.001; MAEs, −8.5–0.8 cm; RMSEs, 0.61–9.94 cm; and CCCs 0.6–1.0; small significant (p < 0.05) bias was present at some sites/timepoints for some measures). Post-hoc analyses revealed potential basis for impact of advancing pregnancy on between-method agreement.

**Conclusions:**

Feasible implementation and accuracy, as shown in the current study, strongly support further development of 3D-optical technology as an alternative to conventional anthropometry for evaluating and monitoring body size, shape, and composition over the course of pregnancy.

## INTRODUCTION

Pregnancy is a dynamic physiological state during which body size and shape change markedly across gestation [1–4]. These pregnancy-associated morphological effects are secondary to fetal and placental growth and related maternal accumulation of adipose tissue, skeletal muscle, and other organs, tissues, and fluids [1, 5, 6]. Variation in the pattern of these changes in maternal body composition is reflected in surface anthropometric measurements throughout pregnancy and are associated with maternal and fetal outcomes [1, 5–10].

Anthropometric assessments commonly used in clinical and research settings include accessible low technology tools and measurements such as scale body weight, stadiometer height, and flexible tape-circumferences [4, 11]. While weight is routinely measured as part of prenatal care, body circumferences are less commonly assessed and may provide additional insight into the physiological changes occurring during this time. Waist circumference, for example, reflects the growing fetus and increasing size of the uterus, but also may reflect changes in adiposity, including visceral adiposity. For example, waist circumferences in our participants is positively correlated with visceral adiposity across gestation as assessed with whole-body magnetic resonance imaging (e.g., at 15, 25, and 35 week evaluations, r values, 0.60, 0.74, and 0.56, all p < 0.001, *personal communication*). Predictions of body fat gain can also be derived from one or more quantified skinfold thicknesses [4–6]. However, these methods and measurements may be time-consuming and, at times impractical, as part of routine prenatal care or within remote health care settings. Acquiring accurate anthropometric body dimensions requires staff training, equipment availability, and attention to routine equipment calibration and maintenance.

Recent advances in digital technology provide a promising opportunity to enhance anthropometric assessment during pregnancy and the postpartum period. Computerized “photogrammetry” methods, first introduced in the 1980s, have evolved rapidly [12]. These methods allow for the creation of three-dimensional (3D) human digital avatars with accurate representations of physical dimensions such as lengths, circumferences, volumes, and surface areas [13]. While early systems were costly and complex, newer 3D scanners are more affordable, portable, and user-friendly [13]. Smartphones equipped with built-in cameras can now acquire both two-dimensional (2D) and 3D images, and available applications can process these data to generate 3D avatars and multiple anthropometric dimensions [14].

These advances, combined with the limitations of traditional anthropometry, led us to conduct a prospective study to evaluate the utility of novel 3D optical technology in quantifying body size and shape during pregnancy. Specifically, we hypothesized that circumferential measures of maternal body size and shape acquired with a 3D optical imaging system would agree closely and correlate significantly with corresponding ground-truth estimates made by a trained anthropometrist with a flexible tape measure.

## METHODS

### Experimental Design and Participants

The current investigation is a secondary analysis of the Mother and Infant NuTrition (MINT) cohort that evaluated body composition across pregnancy and postpartum in persons without diabetes. The primary aim of the MINT cohort was to evaluate adipose tissue changes across pregnancy and postpartum among pregnant individuals with MRI and other body composition measurement tools and is registered through https://www.clinicaltrials.gov/(NCT04132310). MINT was approved by the University of Texas Austin Institutional Review Board and all participants signed an informed consent.

MINT included pregnant individuals at or over the age of 18 years with singleton pregnancies < 14 weeks gestation. Recruitment was through local obstetric providers, social media, and snowball sampling. Exclusion criteria included: body mass index (BMI) < 18.5 or > 35 kg/m^2^, prior history of diabetes, a recognized congenital anomaly, gestational age > 18 weeks, contraindications for the magnetic resonance imaging protocol implemented in the primary phase of the study, history of bariatric surgery, delivery outside of partnered hospitals, regular breastfeeding other infants at the time of baseline evaluation, and recent significant (> 5%) weight change.

All study participants had circumference measurements taken at weeks ~ 15 (range 13–17), ~ 25 (range 23–27), and ~ 35 (range 33–37) of gestation. 3D-optical imaging was performed at each visit along with measurements of waist, hip, mid-upper arm, mid-thigh, and calf circumferences using an inelastic tension-controlled flexible tape (Shorr Canister Tape, Weigh and Measure, LLC, Olney, MD) by a trained staff member. All manual measurements were conducted on the participant’s right side and at least two replicates were measured at each site. If two replicates at the same anatomic location differed by greater than 0.5 cm, a third replicate was obtained. Tape circumference measurements were recorded and averaged.

### Anthropometric Measurements

#### Height and Weight

Participants’ height was measured at the time of enrollment using a stadiometer (Ellard, Monroe, Washington) to the nearest 0.1 cm and weight was measured using a digital scale (Seca 874, Hamburg, Germany) to the nearest 0.1 kg at each of the three visits. Participants were asked to remove shoes and socks prior to height and weight measurements. During height measurement, participants were instructed to keep their feet flat and pointed at a 60° angle with heels, buttocks, and shoulder blades touching the surface of the stadiometer. In addition, participants were requested to look straight ahead while maintaining their head in the Frankfort horizontal plane defined by the lower part of the bony socket containing the eye and the most forward point of the ear. Two replicates of height were recorded. If the two measurements were greater than 0.5 cm apart, a third replicate was collected. Similarly, two weight measurements were recorded during digital scale measurement. If the two weight measurements obtained were greater than 0.5 kg apart, a third replicate was collected.

#### Flexible Tape Circumferences

**Figure 1:**
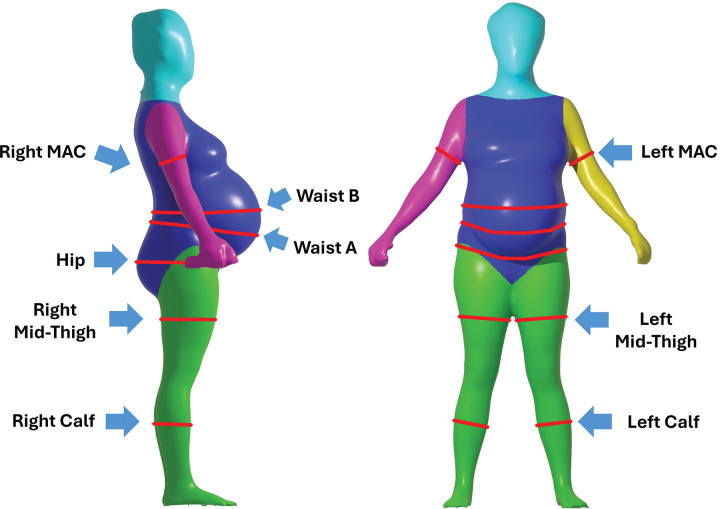
Three-dimensional avatars of a participant at 35-weeks of gestation showing waist (WC), hip, mid-upper arm, mid-thigh, and calf circumference measurement landmarks. Waist circumference A was measured at approximately the same location by the flexible tape and 3D-optical system. Waist circumference B was only measured with the flexible tape. The color pattern identifies body regions (arms, legs, trunk, and head/neck) delineated by the evaluation software.

The five circumferences were measured by trained staff with a flexible measuring tape held snugly and not tightly at each study visit as shown in Fig. 1, a representative participant at 35 weeks of gestation, and are described in detail in the following sections.

##### Waist Circumference.

Waist circumference was measured at the reference anatomic location recommended by the World Health Organization [15]. Participants were first instructed to cross their arms over their chest. Next, the participant’s lowest rib and iliac crest were located. The lowest rib and iliac crest were marked along the mid-axillary line using a washable marker. The distance between the lowest rib and the iliac crest was measured in centimeters to find the midpoint, which was marked. The waist circumference measurement was obtained to the nearest millimeter at the midpoint using the flexible tape measure.

##### Hip Circumference.

Participants were instructed to stand with feet together and arms relaxed by their sides. The maximum extension point of the buttocks was located, and the hip circumference was obtained at this point to the nearest millimeter using the flexible tape measure.

##### Mid-Upper Arm Circumference.

Participants were instructed to stand erect with their arms relaxed by their sides. The midpoint of the arm was located by bending the participant’s elbow to 90° with the palm of the hand facing upward. Next, a trained staff member stood behind the participant to locate the olecranon of the elbow and the lateral tip of the acromion by palpating laterally along the superior surface of the spinous process of the scapula. The halfway point between these two landmarks was located via flexible tape measurement and marked. Using the tape measure, the mid-upper arm circumference was obtained to the nearest millimeter at the denoted halfway point with the participant’s arm relaxed, elbow extended, and palm facing the thigh.

##### Mid-Thigh Circumference.

Participants were instructed to lift the right leg onto a stool about 0.3 m from the ground by flexing their knee and keeping the right foot flat on the surface of the stool. The inguinal crease and proximal border of the patella were located on the right leg, and the distance between the landmarks was obtained to mark the midpoint. The mid-thigh circumference was obtained to the nearest millimeter at the marked midpoint using the flexible tape measure.

##### Calf Circumference.

While maintaining the same position as performed in the mid-thigh circumference evaluation, the participant’s calf circumference was obtained by locating the point of maximum extension of the right calf. Calf circumference was obtained at the point of maximum extension to the nearest millimeter using the flexible tape measure.

### 3D-Optical Imaging

Body circumferences were evaluated at each visit with a 3D-optical scanner, the FIT3D (ProScanner, Redwood City, CA). The Proscanner system has three stationary cameras aligned vertically on a column. The participant stands on a turntable and grasps adjustable handles with arms held in a downward V position, the “A-pose.” The scanner cameras emit a structured light pattern during a 400-degree rotation that is distorted by the participant’s figure; depth is then calculated from the evaluated deformation. Scans require less than one minute to acquire the data needed to reconstruct a 3D avatar from which anthropometric dimensions are acquired. Scanner software generates multiple circumferences and those selected for analysis in this study matched the anatomic sites evaluated with the flexible tape for hip, mid-upper arm, mid-thigh, and calf. Two digital waist circumferences were also selected anticipating the largest “growth” in this region across the different phases of pregnancy, waist circumference A and B. Waist circumference A was measured at the narrowest point of the torso, typically located above the navel and below the ribcage, approximately the same site as the waist circumference measured with the flexible tape. The largest horizontal circumference of the abdomen, usually near the level of the navel, was selected as waist circumference B. Participants were instructed to wear minimal or snug clothing, secure hair back, and remain still during the scan to maintain accuracy. The 3D scans were performed under the supervision of a trained staff member.

### Statistical Methods

The ground-truth flexible tape and 3D digital circumference measurements were compared using descriptive statistics, including group means and standard deviations. Measurements that differed by more than three standard deviations between the two methods were considered outliers and were excluded from the final analysis. The mean absolute errors (MAE, x ± SE) and root-mean square errors (RMSE) were calculated to assess measurement accuracy. Concordance correlation coefficients (CCC) [16] and linear regression analyses (R^2^, p-values) were used to evaluate the agreement and relationships between the ground truth tape and 3D-optical measurements. Bland-Altman analyses [17] were performed to assess measurement biases and agreement limits. Plots of 3D versus flexible tape circumference measurements with corresponding Bland-Altman plots are presented in results for waist A, waist B, and hip circumferences. Comparable plots for mid-upper arm, mid-thigh, and calf circumferences are presented in **Supplementary Information, Figures S1-S3**. Right and left arm and thigh circumference measurements were averaged for analyses and presentation. Statistical analyses were conducted using GraphPad Prism 10 (Boston, MA).

## RESULTS

### Participants

A total of 61 pregnant individuals were enrolled in this phase of the MINT Study. At the initial 15-week visit, 57 participants completed both 3D-optical imaging and flexible tape measurements. Their demographic characteristics are presented in Table 1. Baseline age, height, weight, and BMI were 33 ± 4.3 years, 163.8 ± 7.0 cm, 68.0 ± 11.9 kg, and 25.0 ± 4.0 kg/m^2^, respectively.

Due to research cessation associated with the COVID-19 pandemic, follow-up participation decreased, with 41 participants completing the 25-week visit and 35 completing the 35-week visit. As pregnancy progressed, average weight increased 68.0 ± 11.9 kg at baseline to 74.8 ± 13.5 kg at week 25 and 78.7 ± 12.0 kg at week 35. From baseline to week 25, the average percent change in weight was 10.0%, with an additional 5.2% increase from week 25 to 35.

### 3D-Optical vs. Ground-Truth Tape Measurements

**Figure 2:**
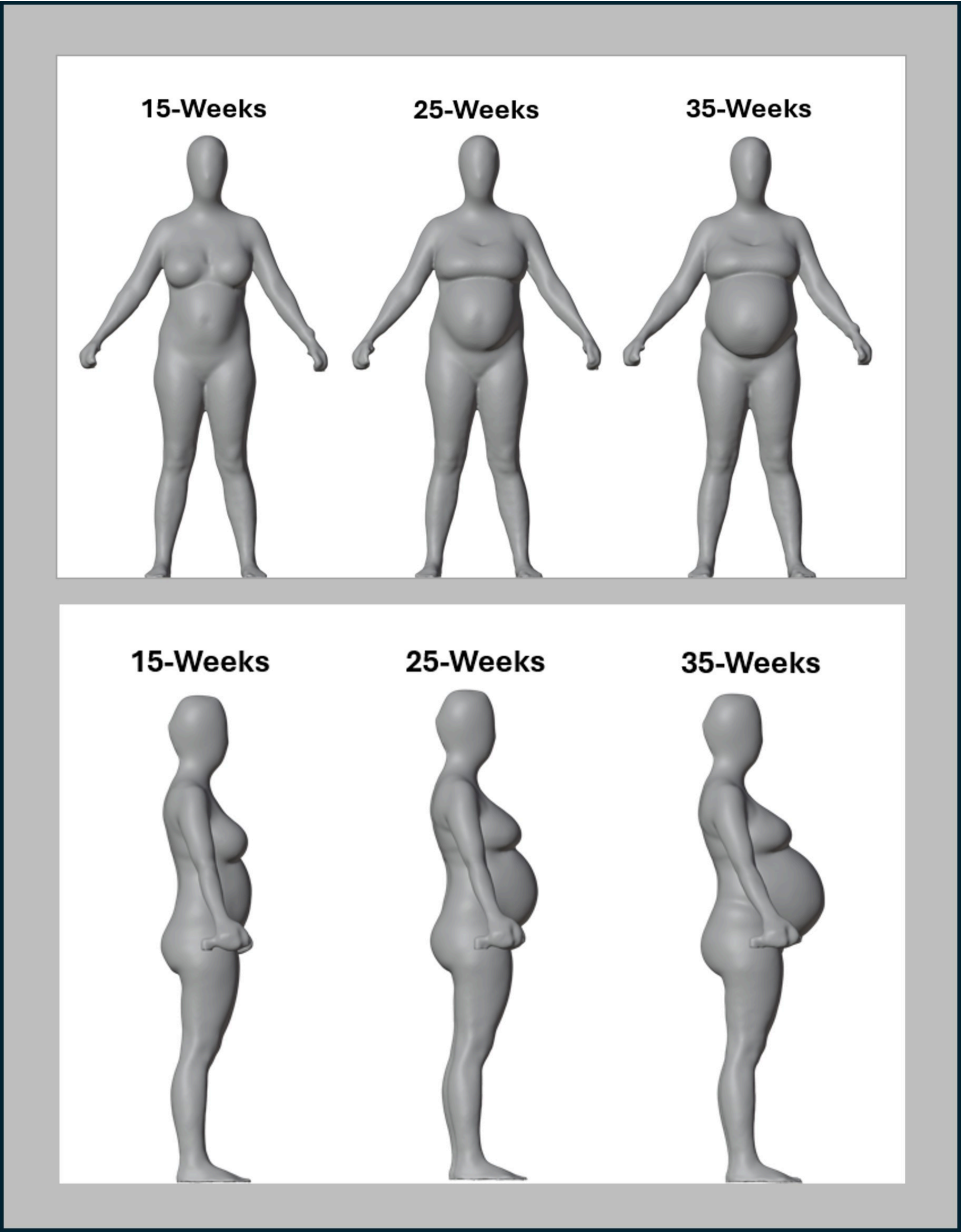
Panel A. Frontal image of a participant’s 3D avatar at gestational weeks 15, 25, and 35. Panel B. Side view of the participant at the evaluation timepoints shown in Panel A.

Circumferences measured by 3D-optical imaging across gestation were significantly correlated with flexible tape measurements, with R^2^ values ranging from 0.63 to 0.97 (all, p < 0.001) and CCCs ranging from 0.5 to 1.0. Three-dimensional images across the evaluated gestational time points are shown in Fig. 2 for a representative participant. Both digital waist circumference measurements (A and B) were compared to the single flexible tape waist circumference measurements.

#### 15-Weeks of Gestation.

**Figure 3:**
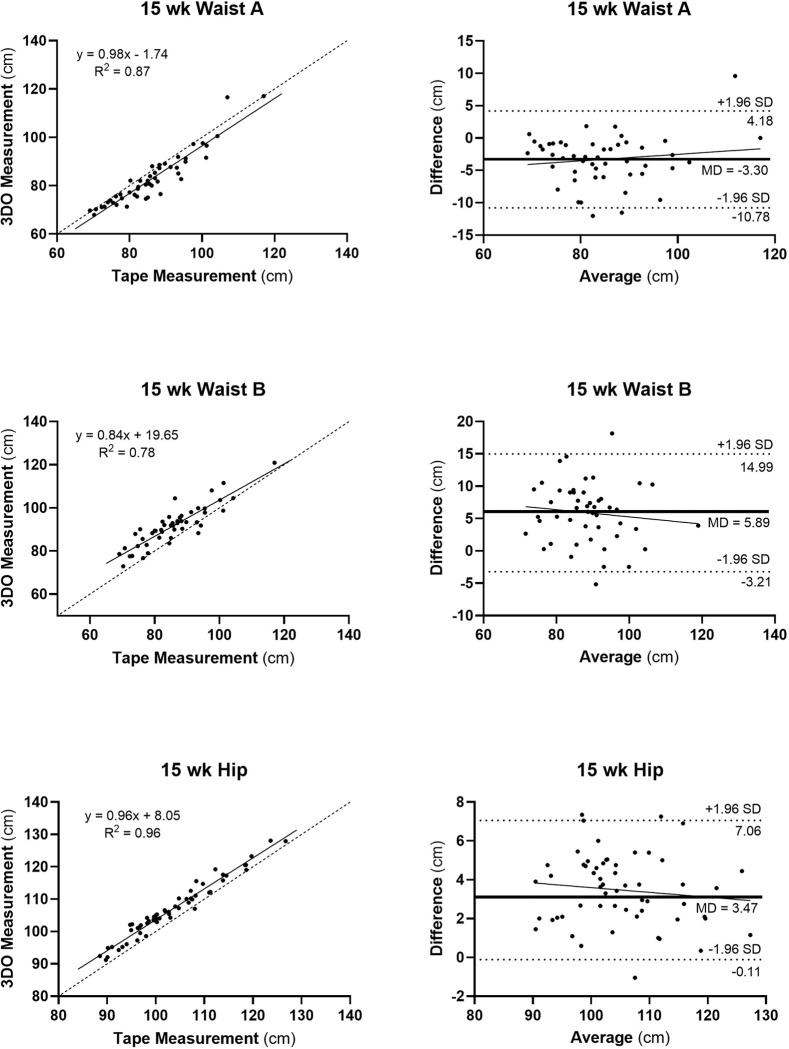
Three-dimensional optical versus flexible tape measurements (left panels) for Waist A, Waist B, and Hip circumferences and corresponding Bland-Altman plots (right panels) at 15-weeks of gestation. Simple linear regression models and R^2^s are shown in each of the plots on the left. All three regression analyses were statistically significant at p<0.001; dashed line is identity. Slopes of Bland-Altman plots were non-significant; dashed lines are mean±1.96 SD and the bold horizontal line is the mean difference (MD) between 3D- and flexible tape-measured circumference, in cm. Mid-upper arm, mid-thigh, and calf circumference plots are shown in **Supplementary Information**.

The strongest agreement was observed in mid-upper arm, mid-thigh, calf, and hip circumferences (e.g., R^2^s ≥ 0.85; RMSE, 0.63–3.89 cm; CCC ≥ 0.9) (Table 2, Fig. 3). Both waist circumference measurements showed slightly lower agreement, particularly for waist B (R^2^, 0.78; RMSE, 7.44 cm; CCC, 0.8) and to a lesser extent waist circumference A (R^2^, 0.87; RMSE, 9.94 cm; CCC, 0.9). The slope of the Bland-Altman plot for mid-arm circumference was non-significant while small significant (p < 0.05) bias was present for mid-thigh and calf circumferences (**Figure S1**). Mean waist circumferences A and B were 3% smaller and 6% larger, respectively, than the ground-truth flexible tape waist circumference measurement.

#### 25-Weeks of Gestation

**Figure 4:**
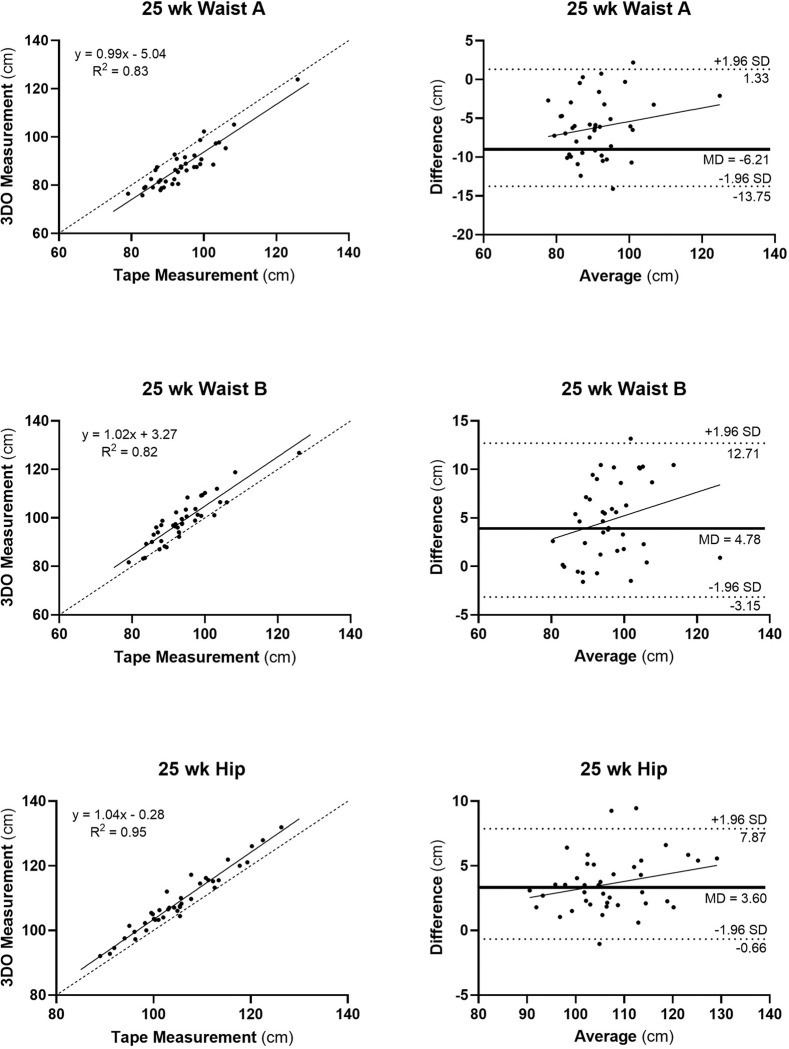
Three-dimensional optical versus flexible tape measurements (left panels) for Waist A, Waist B, and Hip circumferences and corresponding Bland-Altman plots (right panels) at 25-weeks of gestation. Simple linear regression models and R^2^s are shown in each of the plots on the left. All three regression analyses were statistically significant at p<0.001; dashed line is identity. Slopes of Bland-Altman plots were non-significant; dashed lines are mean±1.96 SD and the bold horizontal line is the mean difference (MD) between 3D- and flexible tape-measured circumference, in cm. Mid-upper arm, mid-thigh, and calf circumference plots are shown in **Supplementary Information**.

Most circumferences retained strong agreement between 3D and flexible tape measurements (Table 2, Fig. 4). Hip, mid-thigh, and calf circumferences remained highly consistent between digital and tape methods (e.g., R^2^ ≥0.92; RMSE, 0.61–4.19 cm; CCC, 0.9–1.0), while mid-arm circumference agreement decreased (from R^2^, 0.92; RMSE, 0.99 cm; CCC, 1.0 to R^2^, 0.76; RMSE, 1.75; CCC, 0.8). Both digital waist circumferences A and B correlations with flexible tape measurements remained strong (R^2^, 0.82 and 0.83, respectively) with agreement slightly lower than for hip, mid-thigh, and calf circumferences (R^2^, 0.95, 0.92 and 0.97, respectively). The slope of the Bland-Altman plot for mid-arm circumference was non-significant while small significant (p < 0.05) bias was again present for mid-thigh and calf circumferences (**Figure S2**). Waist A and B were 7% smaller and 5% larger than the ground-truth waist circumference measurements.

#### 35-weeks of Gestation.

**Figure 5:**
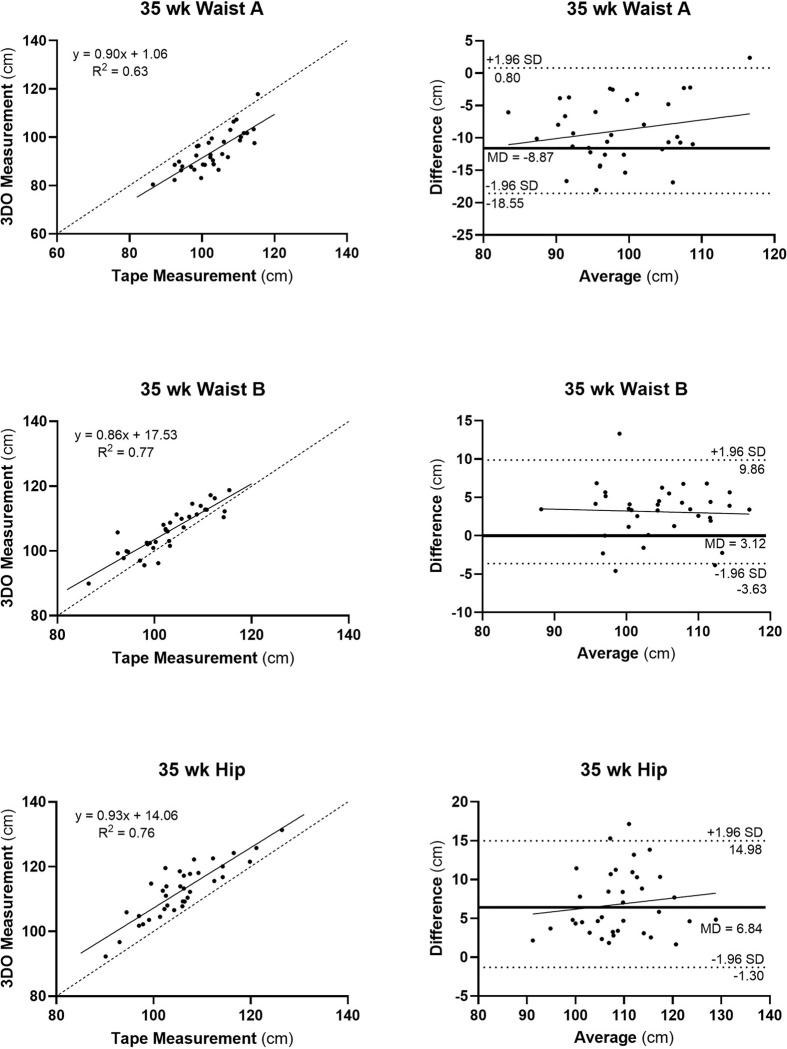
Three-dimensional optical versus flexible tape measurements (left panels) for Waist A, Waist B, and Hip circumferences and corresponding Bland-Altman plots (right panels) at 35-weeks of gestation. Simple linear regression models and R^2^s are shown in each of the plots on the left. All three regression analyses were statistically significant at p<0.001; dashed line is identity. Slopes of Bland-Altman plots were non-significant; dashed lines are mean±1.96 SD and the bold horizontal line is the mean difference (MD) between 3D- and flexible tape-measured circumference, in cm. Mid-upper arm, mid-thigh, and calf circumference plots are shown in **Supplementary Information**.

Mid-thigh and calf circumferences maintained strong agreement between flexible tape and ground truth measurements (R^2^ ≥0.85; RMSE, 0.72–1.60 cm; CCC, 0.9–1.0) (Table 2, Fig. 5). Mid-arm circumference agreement improved minimally from the 25-week measurements (from R^2^, 0.76; RMSE, 1.75 cm; CCC, 0.8 to R^2^, 0.79; RMSE, 1.44 cm; CCC, 0.9). Agreement between digital hip, waist A, and waist B circumferences with flexible tape measurements decreased from the 25-week evaluation (R^2^, 0.82–0.95; RMSE, 4.19–7.28 cm; CCCs, 0.7–0.9 to R^2^, 0.63–0.77; RMSE, 4.61–10.12 cm; CCC, 0.5–0.8), with waist circumference A agreement lowest overall (R^2^, 0.83; 7.28 cm; CCC, 0.7 to R^2^, 0.63; RMSE, 10.12 cm; CCC, 0.5). The slopes of all Bland-Altman plots were non-significant (**Figure S3**). Waist circumferences A and B were 8% smaller and 3% larger than the ground-truth waist circumference measurement.

#### Composite Associations Across Gestation.

**Figure 6:**
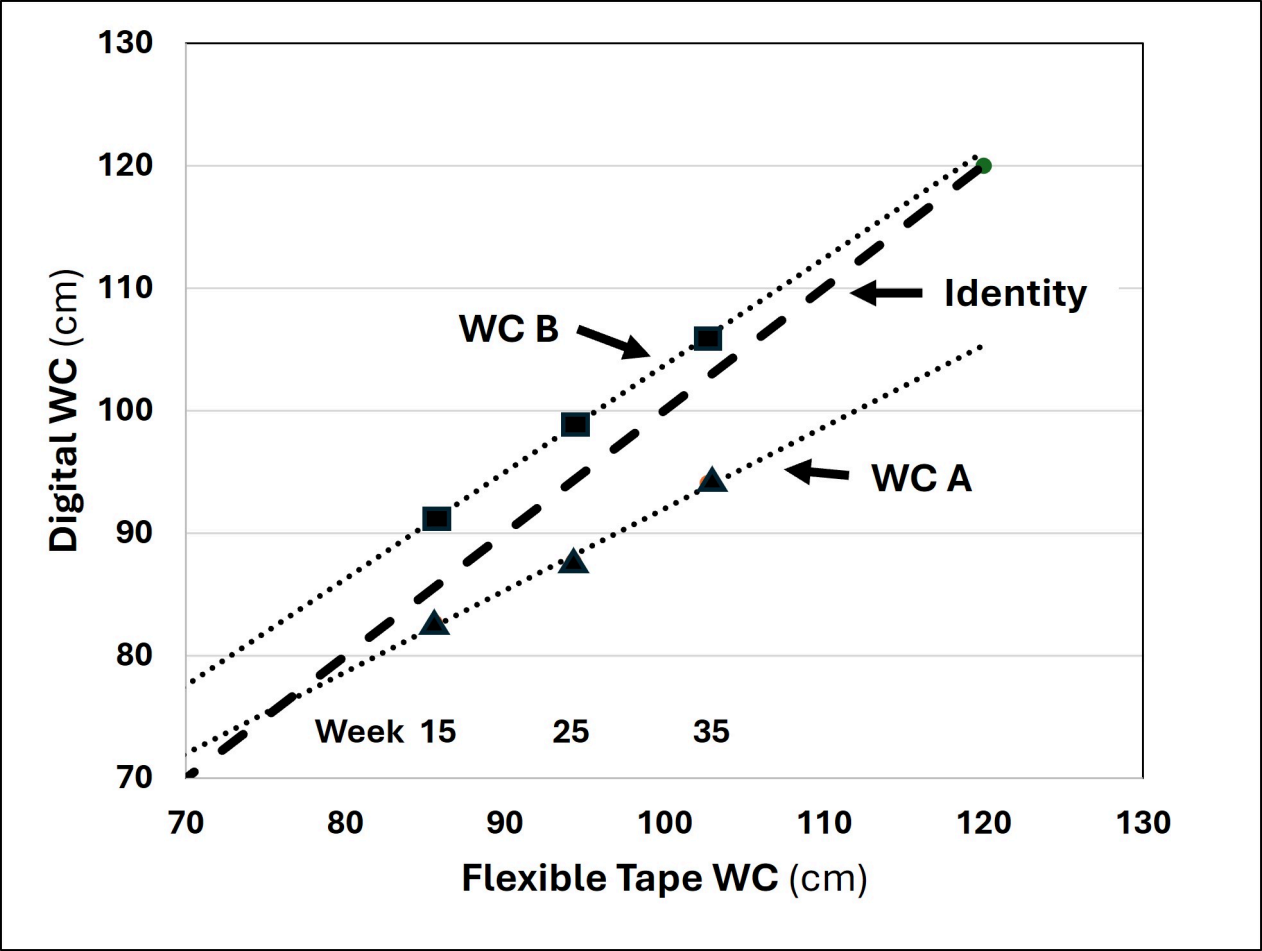
Digital waist circumferences (WC) A and B versus flexible tape waist circumference measurement at 15-, 25-, and 35-week evaluations. Regression lines are fit to the data points presented that represent the group mean values shown in Table 2. Bold dash line is identity.

While overall agreement between 3D and flexible tape circumference measures remained strong across the three gestational evaluations, a pattern emerged among R^2^, MAE, RMSE, and CCC as described in Table 2. Viewed across gestation, each of the four measurements of agreement tended to show the poorest performance at week 35 for two central body circumferences, waist A and hip. By contrast, corresponding values for the central digital waist circumference B tended to improve over the three evaluation time points. A relevant pattern of these relations is shown in Fig. 6 with a plot of digital waist A and B circumferences against ground-truth flexible tape waist measurements at 15, 25, and 35 weeks. While the mean value of waist circumference A agreed well with flexible tape measurements at 15 weeks, progressively less agreement was present at 25 and 35 weeks of gestation. An almost identical but opposite pattern was present for waist circumference B with flexible tape agreement improving over the course of pregnancy. The same pattern of change over time was present for hip circumference as for waist circumference A (i.e., deviation over time from ground-truth estimates). Agreement between 3D and flexible tape measurements for the remaining evaluated circumferences, mid-upper arm, mid-thigh, and calf tended to be reasonably stable, with small but statistically significant bias present for mid-thigh and calf circumferences at 15 and 25 weeks of gestation.

## DISCUSSION

The current study aimed to evaluate the hypothesis predicting that body circumferences evaluated with a 3D-optical system would agree closely and correlate significantly with corresponding ground-truth estimates provided by flexible tape measurements made by trained staff across the dynamic phases of pregnancy. Our hypothesis was largely supported: overall, 3D-optical circumference measurements agreed closely with ground-truth estimates as defined by multiple metrics (R^2^, MAE, RMSE, CCC, and Bland-Altman plots), strongly supporting further development of safe, practical, and relatively low-cost 3D imaging technology for clinical applications across pregnancy.

While good overall agreement between 3D-optical and ground-truth measurements was observed, clear patterns emerged across the three evaluation time points: strong and consistent agreement, with some small bias, was present at all three measurement time points for peripheral circumferences (mid-upper arm, mid-thigh, and calf); and, agreement diminished during advancing pregnancy for central body-waist A and hip circumferences even though agreement improved for central body-waist circumference B. These patterns, which emerged during analysis of the current study findings, has a plausible explanation. Our working theory is that the digital central circumferences measured by system software were made at the identical landmarked anatomic sites independent of pregnancy status. By contrast, the expanding central maternal girth over time likely subtly moved the “reference” anatomic sites evaluated with the flexible tape by trained staff. That is, the waist circumference site measured with the tape may have migrated from its “standard location”, thus moving it away from digital waist circumference A and closer to waist circumference B. This suggestion is supported by the pattern of 15-, 25-, and 35-week waist circumference measurements made with the flexible tape and 3D-optical system shown in Fig. 6. A similar effect, movement away from its reference site over the course of pregnancy, likely occurred for the hip circumference measurements. If our hypothesis is correct, these findings do not reveal a flaw in 3D-optical technology but rather an intrinsic difference in how trained staff and digital software quantify body dimensions during the dynamic stages of pregnancy during which the central body markedly enlarges and bony landmarks may subtly shift with increasing gestational age. These conjectures can be further evaluated in future studies.

The focus of the current study was to evaluate specific body circumferences, a key to signaling the potential for not only additional anthropometric measurements (e.g., lengths, volumes, and surface areas), but for deriving accurate evaluation of body composition. The acquired 3D-optical circumferences and potentially other anthropometric measurements could be used to derive estimates of maternal body fat, skeletal muscle, and related clinical outcomes. Additionally, circumferences evaluated with 3D-optical systems could be combined with skinfold measurements to further improve body composition predictions. Studies such as these are now increasingly feasible as cost for 3D-optical systems declines and availability of devices increases as other low-cost imaging technology improves [14].

### Limitations

Several limitations were present in the current study and with the evaluated 3D imaging technology. Given that the study was designed to assess body composition with multiple methods, including MRI, we were only able to include individuals with BMI categories in the healthy, overweight and class I obesity range. Thus, our findings may not be generalizable to those with pre-pregnancy underweight or pre-pregnancy obesity class II or III. Our sample size decreased over time due to the COVID-19 pandemic and thus reduced the sample size at later gestational visits, potentially limiting generalizability. As noted earlier, flexible tape measurements were used as the ground-truth reference standard, although this human-guided measurement approach is not without concerns, particularly for dynamic central-body regions of growth such as the waist and hip. Laser technology that can capture high resolution 3D images [18] could potentially be used as the ground-truth measurement device in future studies while maintaining identical landmarks to less costly but more available imaging devices across pregnancy. The 3D imaging technology requires some space for optimum performance, hence clinical applications might be constrained in some facilities by this requirement. Lastly, optimum image capture is facilitated by participants clothed in tight fitting garments, a requirement not always feasible for some individuals and in some settings.

### Future Opportunities

Digital 3D-optical systems often include software that generates hundreds of body surface measurements that could be used along with analysis methods such as artificial intelligence to predict not only body composition, but clinical outcomes [19]. In support of that prediction, many studies report links between anthropometric measurements, for example mid-upper arm circumference, with excessive weight gain and risk of gestational diabetes [20, 21], malnutrition [22], and intrauterine growth restriction, increased risk of preterm birth, and small for gestational age [8, 23]. Identifying these insalubrious outcomes is a high clinical priority for which 3D-optical imaging might serve a useful role.

## Conclusion

The current study extends earlier observations in non-pregnant adults [9, 10] supporting the accuracy of 3D-optical imaging in quantifying selected body circumferences across three key timepoints in pregnant individuals. Our findings pave the way for future studies to expand on these observations with collection of additional anthropometric measurements that associate these estimates with body composition and clinical outcomes. The observations reported herein also suggest that the locations of “standard” anatomic sites, such as the waist, may be more fluid during pregnancy than previously recognized. Safe, practical, and relatively inexpensive, 3D-optical imaging may offer a new opportunity beyond traditional anthropometric measurements when evaluating and monitoring pregnant individuals.

## Supplementary Material

Supplementary Files

This is a list of supplementary files associated with this preprint. Click to download.

• Mintdigitalanthropaper81425SupplementalInformation.docx

## Figures and Tables

**Table 1:** Participant baseline characteristics (n = 57).

Characteristic	n (%)
Ethnicity	
Hispanic/Latina	9 (16)
Not Hispanic/Latina	48 (84)
Race	
American Indian or Alaska Native	4 (7)
Asian	6 (10)
Black or African American	3 (5)
White	50 (88)
Multiracial	5 (9)
Unknown	1 (2)
Parity (> 32 weeks)	
0	35 (61)
1	16 (28)
2	6 (11)
Marital Status	
Married	52 (91)
Not married & living with significant other	3 (5)
Not married	2 (4)
Annual Income	
< $75,000	7 (12)
$75-$100,000	8 (14)
>$100,000	42 (74)
Education	
No College	2 (3)
Some College, Business or Technical School	1 (2)
College Degree	29 (51)
Post Graduate Work	25 (44)
Pre-pregnancy BMI Category	
Healthy (BMI < 25)	36 (63)
Overweight or obesity (BMI ≥ 25)	21 (37)

BMI, body mass index; SD, standard deviation.

**Table 2:** Results of circumference evaluations at the three evaluation timepoints.

Circumference	Week	Ground-Truth (mean ± SD, cm)	3D (mean ± SD, cm)	MAE ± SD (cm)	RMSE (cm)	R^2^*	CCC
Waist A	15	85.6 ± 10.0^1^	82.7 ± 10.3	−8.5 ± 5.0	9.94	0.87	0.9
	25	94.3 ± 8.7^1^	87.7 ± 9.2	−6.1 ± 3.8	7.28	0.83	0.7
	35	102.7 ± 7.1^1^	94.1 ± 8.0	−8.6 ± 4.9	10.12	0.63	0.5
Waist B	15	85.6 ± 10.0^1^	91.2 ± 9.1	−5.9 ± 4.5	7.44	0.78	0.8
	25	94.3 ± 8.7^1^	98.7 ± 9.5	4.7 ± 4.0	6.23	0.82	0.8
	35	102.7 ± 7.1^1^	106.1 ± 6.9	3.0 ± 3.4	4.61	0.77	0.8
Hip	15	103.3 ± 8.8^1^	106.8 ± 8.8	3.3 ± 1.8	3.89	0.96	0.9
	25	105.2 ± 8.7^1^	108.8 ± 9.3	3.5 ± 2.2	4.19	0.95	0.9
	35	105.7 ± 7.9^1^	112.6 ± 8.4	6.7 ± 4.2	7.97	0.76	0.6
Mid-Arm	15	29.8 ± 3.4^1^	30.1 ± 3.4	0.3 ± 1.0	0.99	0.92	1.0
	25	29.8 ± 3.2^1^	30.4 ± 3.1	0.8 ± 1.6	1.75	0.76	0.8
	35	30.1 ± 2.9^1^	30.6 ± 2.9	0.5 ± 1.4	1.44	0.79	0.9
Mid-Thigh	15	48.6 ± 4.1^2^	48.8 ± 4.3	−0.2 ± 1.9	1.92	0.85‡	0.9
	25	49.2 ± 4.9^2^	49.1 ± 4.2	−0.1 ± 1.6	1.56	0.92‡	1.0
	35	49.6 ± 4.2^2^	49.5± 3.9	−0.1 ± 1.6	1.60	0.85	0.9
Calf	15	36.8 ± 3.1	37.1 ±2.9	0.3 ± 0.6	0.63	0.97‡	1.0
	25	37.4 ± 3.2^1^	37.5 ± 3.0	0.2 ± 0.6	0.61	0.97‡	1.0
	35	37.8 ± 2.6	37.9 ± 2.7	0.1 ± 0.7	0.72	0.93	1.0

1 p < 0.05 for comparison of mean circumferences

2 indicates findings were non-significant;

* all regression analyses shown in figures were p < 0.001;

‡ indicates Bland-Altman slope signifi cant at p < 0.05. Sample sizes at 15, 25, and 25 weeks were 57, 41, 35, respectively. Abbreviations: CCC,concordance correlation coefficient; GT, ground-truth; HC, hip circumference; L, left; MAE, meanabsolute error; Pred, predicted; R, right; RMSE, root-mean square error; WC, waist circumference.

## Data Availability

Data described in the manuscript will be made available upon reasonable request pending application and approval by the investigators.

## Abbreviations

3D: three-dimensional
3DO: 3D-optical
2D: two-dimensional
BMI: body mass index
CCC: concordance correlation coefficient
MAE: mean absolute error
MD: mean difference
MINT: Mother Infant NuTrition
RMSE: root-mean square error
SD: standard deviation
SE: standard error
